# Isorhamnetin Has Potential for the Treatment of *Escherichia coli*-Induced Sepsis

**DOI:** 10.3390/molecules24213984

**Published:** 2019-11-04

**Authors:** Anil Kumar Chauhan, Jieun Kim, Yeongjoon Lee, Pavithra K. Balasubramanian, Yangmee Kim

**Affiliations:** Department of Bioscience and Biotechnology, Research Institute for Bioactive-Metabolome Network, Konkuk University, Seoul 05029, Korea; chauhananil48@konkuk.ac.kr (A.K.C.); za3524@konkuk.ac.kr (J.K.); lyj7956@konkuk.ac.kr (Y.L.); Pavithra@konkuk.ac.kr (P.K.B.)

**Keywords:** isorhamnetin, flavonoid, bacterial sepsis, toll-like receptor 4, inflammation

## Abstract

Isorhamnetin is a flavonoid that is abundant in the fruit of *Hippophae rhamnoides* L. It is widely studied for its ability to modulate inflammatory responses. In this study, we evaluated the potential of isorhamnetin to prevent gram-negative sepsis. We investigated its efficacy using an *Escherichia coli*-induced sepsis model. Our study reveals that isorhamnetin treatment significantly enhances survival and reduces proinflammatory cytokine levels in the serum and lung tissue of *E. coli*-infected mice. Further, isorhamnetin treatment also significantly reduces the levels of aspartate aminotransferase, alanine amino transferase and blood urea nitrogen, suggesting that it can improve liver and kidney function in infected mice. Docking studies reveal that isorhamnetin binds deep in the hydrophobic binding pocket of MD-2 via extensive hydrophobic interactions and hydrogen bonding with Tyr102, preventing TLR4/MD-2 dimerization. Notably, binding and secreted alkaline phosphatase reporter gene assays show that isorhamnetin can interact directly with the TLR4/MD-2 complex, thus inhibiting the TLR4 cascade, which eventually causes systemic inflammation, resulting in death due to cytokine storms. We therefore presume that isorhamnetin could be a suitable therapeutic candidate to treat bacterial sepsis.

## 1. Introduction 

Inflammation is an innate immune response against microbial infection. However, if it persists for a long time, it can cause several fatal diseases, including sepsis. Sepsis is a clinical condition defined as a systemic inflammation in the body in response to microbial infection, which eventually leads to multiple organ failure. Sepsis is reported to be the leading cause of mortality across the globe, and thus the World Health Organization (WHO) devoted the year 2017 to prioritize this disease and its treatment strategies [1,2,3]. The organs and systems that are commonly affected by sepsis include the lung, abdomen, blood and kidneys, with associated incidences of 64%, 20%, 15% and 14%, respectively [3]. Pathophysiological studies also reveal that the activation of toll-like receptors (TLRs) by microbes or microbial peptides represents the principle mechanism underlying the development of sepsis [3,4].

TLRs are transmembrane receptors present in major immune cells, and are composed of an extracellular leucine-rich repeat domain, a transmembrane region and an intracellular toll-interleukin-1 receptor (TIR) domain [5,6]. 

In total, 10 and 12 TLRs are identifiable in human beings and mice, respectively, and each of them recognizes specific pathogen-associated molecular patterns [7]. However, among these TLRs, TLR4 is responsible for the recognition of bacterial lipopolysaccharide (LPS) and the initiation of an immune response against LPS and/or gram-negative bacteria. Notably, TLR4 requires MD-2 to recognize LPS, and a TLR4–MD-2–LPS complex is essential to activate the TLR4 pathway in macrophages. Once TLR4 recognizes LPS, it undergoes an oligomerization process to recruit downstream adaptor molecules via interactions with the TIR domain. Five adaptor proteins that contain the TIR domain are identifiable, including MyD88 (myeloid differentiation primary response gene 88), TIRAP (TIR domain-containing adaptor protein, also known as Mal, MyD88-adapter-like), TRIF (TIR domain-containing adaptor inducing IFN-β), TRAM (TRIF-related adaptor molecule), and SARM (sterile α and HEAT-Armadillo motifs-containing protein) [8]. These adaptor proteins play crucial roles in the TLR4 pathway, which eventually results in the translocation of NF-κB from the cytoplasm to the nucleus to trigger the production of pro-inflammatory cytokines [7]. 

**Figure 1:**
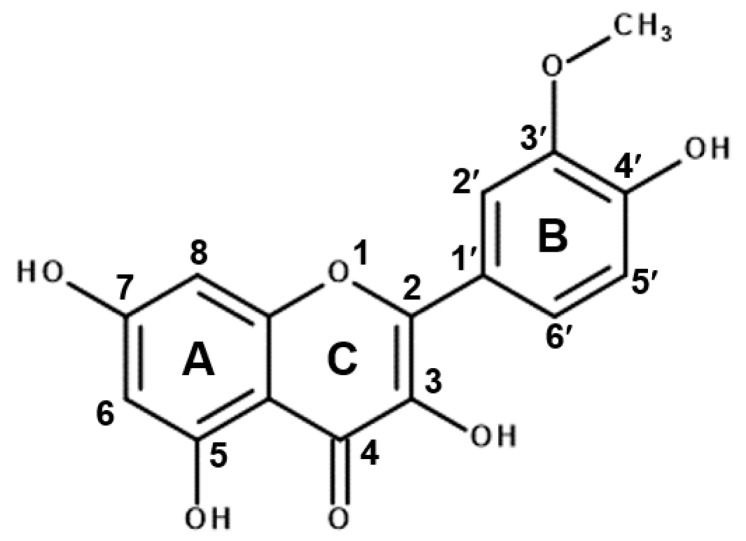
Chemical structure of isorhamnetin also known as 3′-methoxyquercetin (molecular weight: 316.26). The image was drawn using Chemdraw software.

Flavonoids are naturally-occurring polyphenols that are found in a variety of edible plants. Chemically, they exist in glycosylated (conjugated with sugar) or aglycone forms (free form) [9]. The role of flavonoids in plants is crucial, as they confer infection resistance to the plant [10], in addition to protecting them from harmful ultraviolet rays [10,11]. Moreover, much research was carried out to explore the role of flavonoids in animal systems, and among the various activities shown by all types of flavonoids, their antioxidant potential is the most notable [12]. They are also reported to have other health-promoting effects, such as anti-inflammatory activity. For example, the cytoprotective efficacy of plant flavanol quercetins is widely explored [13,14]. Isorhamnetin (Figure 1), another plant flavanol, is a 3′-O-methylated metabolite of quercetin, and is found predominantly in the fruit of *Hippophae rhamnoides* L.; it exerts various biological effects, including anti-inflammatory [15], anticancer [16] and antioxidant activities [17]. Further, we previously reported that this flavonoid has potent anti-tuberculosis activity against *Mycobacterium tuberculosis* H37Rv and multi-drug- and extensively drug-resistant clinical isolates [18]. Importantly, the absorption and metabolic stability of methylated flavonoids are believed to be higher than those of unmethylated flavanols [16]. Thus, this property makes isorhamnetin more potent than quercetin. 

In the present study, we explored the potential of isorhamnetin to protect against *Escherichia coli*-induced sepsis by establishing a murine model. Furthermore, based upon binding affinity and a molecular docking examination, we show for the first time, to our knowledge, that isorhamnetin can bind TLR4/MD-2 directly, thus preventing the activation of the TLR4 cascade, which is responsible for sepsis progression. The outcomes of our study might provide further insights into the natural flavonoid isorhamnetin and its role in sepsis prevention and treatment. 

## 2. Results

### 2.1. Isorhamnetin Treatment Protects Mice from E. coli-Induced Sepsis 

**Figure 2:**
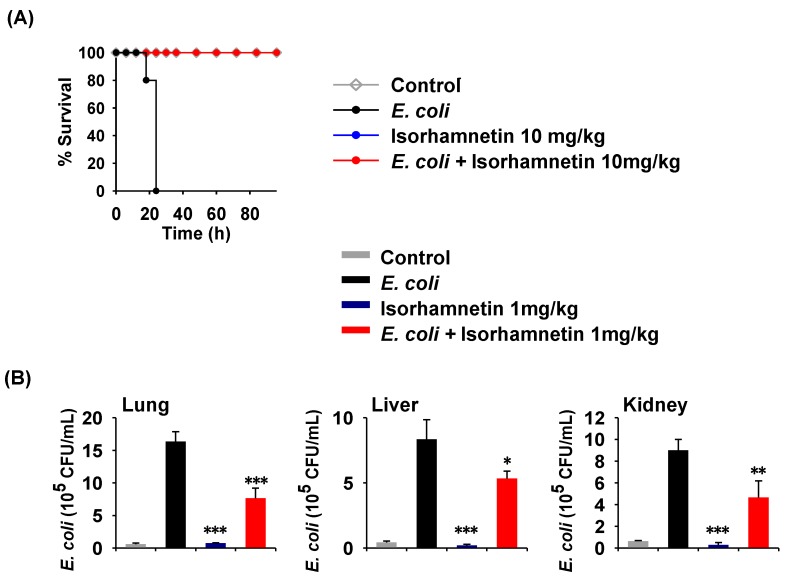
In vivo examination of the effects of isorhamnetin on *Escherichia coli*-induced sepsis. (**A**) Potential of this compound to promote the survival of mice in response to *E. coli*-induced sepsis. (**B**) Evaluation of the bacterial load in the visceral organs of mice. Data are presented as the means ± standard error of the mean (SEM). * *p* < 0.05; ** *p* < 0.01; *** *p* < 0.001 compared to the *E. coli* group.

It was previously reported that isorhamnetin can reduce inflammation by downregulating nitric oxide (NO) and cytokine production [19]. Therefore, we sought to examine the ability of isorhamnetin to protect mice from *E. coli*-induced sepsis. To establish a bacterial sepsis model, we used a virulent *E. coli* K1 strain to infect isorhamnetin-pretreated mice for 96 h for survival assays and 18 h to examine inflammation. As depicted in Figure 2A, mice in the isorhamnetin-treated group survived until the end of the experiment, but the *E. coli*-treated mice died within 24 h of infection. Further, we assessed the bacterial burden in the visceral organs of the mice (lung, liver, and kidneys) and found that isorhamnetin treatment significantly reduced the bacterial population in all organs compared to that in the *E. coli* only group (Figure 2B). Although isorhamnetin has no antibacterial activity in vitro, it is reported to enhance macrophage functions, such as phagocytosis, by increasing superoxide generation after the engulfment of bacteria [20]. Thus, we speculate that the reduction in bacterial loads in the organs is a result of the immunomodulatory potential of isorhamnetin.

### 2.2. Isorhamnetin Treatment Protects Mice from E. coli-Induced Inflammation

**Figure 3:**
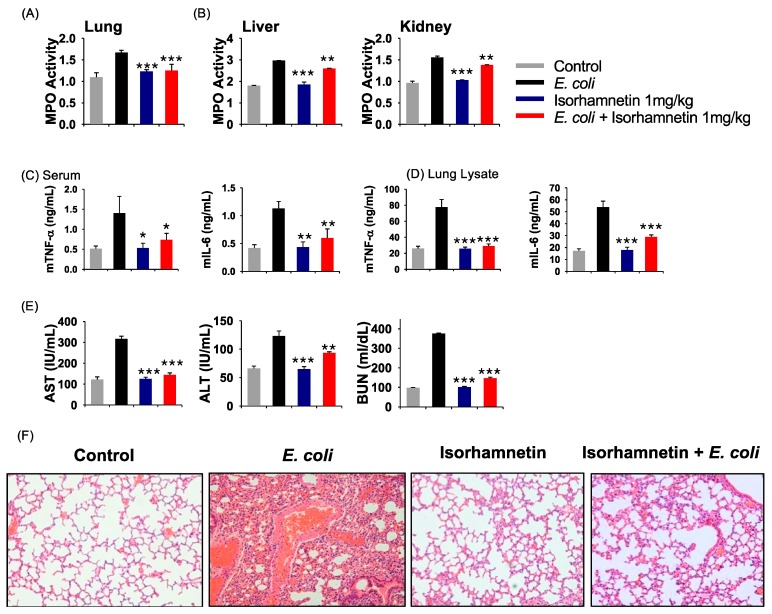
In vivo anti-inflammatory and toxicity evaluation of isorhamnetin. (**A**,**B**) Examination of myeloperoxidase (MPO) activity in lung, liver and kidney. (**C**,**D**) Production of cytokines (TNF-α, IL-6) in the sera and lung lysates. (**E**) Aspartate aminotransferase (AST), alanine amino transferase (ALT) and blood urea nitrogen (BUN) levels in the serum. (F) Hematoxylin and eosin staining of the lung tissue. Data are presented as the means ± SEM. * *p* < 0.05; ** *p* < 0.01; and *** *p* < 0.001 compared to the *E. coli* group.

The effect of isorhamnetin on *E. coli*-induced myeloperoxidase (MPO) and proinflammatory cytokines (TNF-α and IL-6) was then examined. As depicted in Figure 3A,B, MPO activity is high in untreated mice infected with *E. coli*, whereas treatment with isorhamnetin decreased MPO activity in the kidney, liver and lung. However, the best results were obtained in the lung tissue (Figure 3B). Moreover, the secretion of cytokines is also highly increased in the sera and lung lysates after *E. coli* infection, which was drastically reduced in the isorhamnetin-treated group, suggesting that isorhamnetin can suppress the inflammatory response (Figure 3C,D). 

Additionally, aspartate aminotransferase (AST), alanine amino transferase (ALT) and blood urea nitrogen (BUN) assays were carried out to evaluate liver and kidney function, and the results of these assays reveal that untreated mice infected with *E. coli* develop severe liver and kidney dysfunction (Figure 3E). However, isorhamnetin treatment protects the mouse liver and kidneys from *E. coli*-induced inflammation, as indicated by the reduced levels of AST, ALT and BUN (Figure 3E). Notably, we also examined the effect of isorhamnetin on uninfected mice to assess toxicity; here, we observed that this treatment does not alter the levels of AST, ALT and BUN, compared to those in the control group (Figure 3E), clearly indicating its safety. Moreover, we examined the histopathology of the lung tissues to assess neutrophil infiltration; the results indicate that isorhamnetin treatment could reduce the infiltration of neutrophils in the lung, which is noticeable after *E. coli* treatment (Figure 3F).

### 2.3. Interactions between Isorhamnetin and MD-2 Represent the Principal Mechanism Underlying the Anti-Inflammatory Activity

As we observed that isorhamnetin could successfully promote the survival of *E. coli*-infected mice and ameliorate inflammation, we next determined its probable mechanism of action. Isorhamnetin was previously reported to downregulate the TLR4 pathway [21], which has a crucial role in the development of inflammation. We therefore measured the binding affinity of isorhamnetin to MD-2 by surface plasmon resonance (SPR), as it is reported that MD-2 is responsible for binding hydrophobic LPS and other small molecules [22], which eventually induces the dimerization of TLR4/MD-2. 

**Figure 4:**
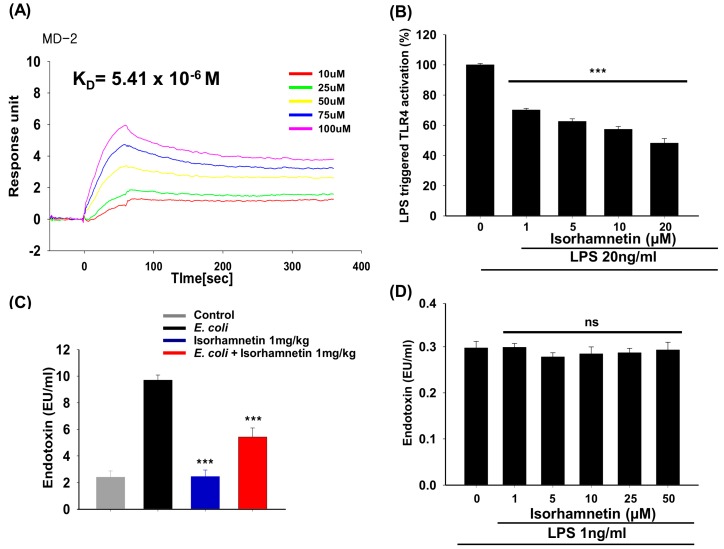
Effect of isorhamnetin on binding to MD-2, LPS-induced SEAP secretion and LPS neutralization (**A**) Surface plasmon resonance (SPR) analysis to determine the binding interaction. (**B**) Secretary alkaline phosphate (SEAP) assay in HEK-Blue™ hTLR4 cells (toll-like receptors (TLRs)). (**C**) Limulus amebocyte lysate (LAL) assay in serum of mice. (**D**) LAL assay to detect LPS neutralization in vitro.

The binding affinity of isorhamnetin for MD-2 is very strong, specifically 5.41 × 10^−6^ M (association rate: 2.296 × 10^2^ M^−1^ s^−1^; dissociation rate: 1.242 × 10^−3^ s^−1^; Figure 4A).

Furthermore, we performed a SEAP (secreted embryonic alkaline phosphatase) assay using HEK-Blue™-hTLR4 cells to confirm the selectivity of isorhamnetin for the TLR4 receptor. SEAP is a protein secreted after the translocation of NF-κB from the cytosol to the nucleus; the amount of SEAP secreted into the medium correlates with the activation of the TLR4 pathway. Results of this experiment reveal that isorhamnetin could reduce SEAP activity in a concentration-dependent manner, with a 52% inhibition rate, indicating its specificity for the TLR4 receptor (Figure 4B).

We also carried out a Limulus amebocyte lysate (LAL) assay to examine the level of LPS in the mice serum and the in vitro LPS neutralizing efficacy of isorhamnetin. As, depicted in Figure 4C the level of LPS in the *E. coli* treatment group is significantly higher than those of the isorhamnetin treatment group (*p* < 0.001), suggesting that isorhamnetin treatment causes the reduction of the *E. coli* count, thus the level of LPS is decreased. However, the in vitro assessment of LPS neutralization reveals that isorhamnetin has no effect on the neutralization of LPS, as the level of LPS is similar in both the isorhamnetin treatment group as well as the LPS-only-treated group (Figure 4D), implying that isorhamnetin does not neutralize LPS, and instead it may give a competition to LPS in binding with MD-2.

### 2.4. Molecular Docking

**Figure 5:**
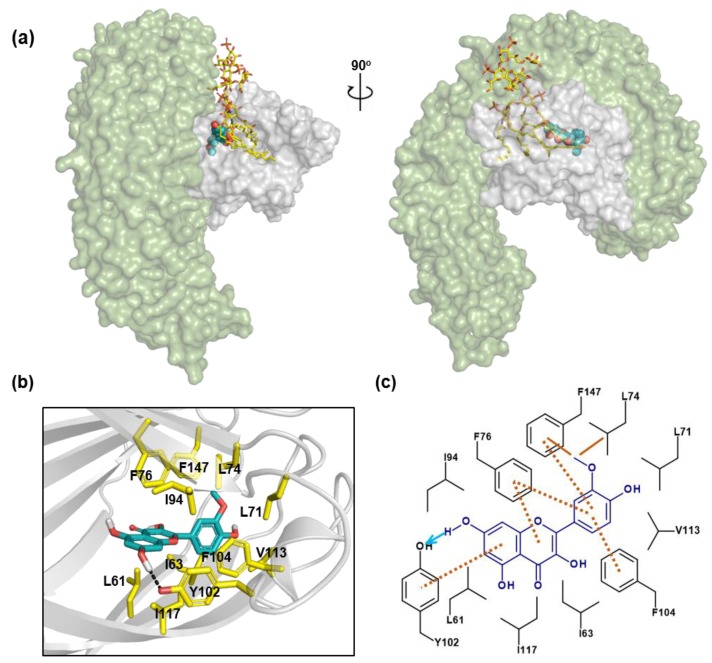
Binding interactions between isorhamnetin and MD-2. (**a**) Isorhamnetin (blue and red sphere) overlapped with LPS (yellow stick) in the hydrophobic binding cavity of MD-2 (gray) together with TLR4 (green), and its 90-degree rotated complex structure is shown. (**b**) Overview of the MD-2–isorhamnetin complex. Isorhamnetin is shown as sky blue sticks, and important residues in MD-2 (gray ribbon cartoon) are shown as yellow sticks. (**c**) Two-dimensional illustration of the MD-2–isorhamnetin docking pose showing hydrophobic interactions and hydrogen bonding interactions.

To understand the interaction between isorhamnetin and MD-2 at the molecular level, a binding model of isorhamnetin to MD-2 was determined by a docking simulation. A model of the binding between isorhamnetin and MD-2 shows that isorhamnetin was inserted into the hydrophobic binding pocket of MD-2, which is a part of the LPS-binding site (Figure 5a). Isorhamnetin also exhibits extensive hydrophobic interactions with MD-2. Further, the 3′-OCH3 of isorhamnetin bound to the deep hydrophobic pocket of MD-2, forming hydrophobic interactions with Leu74 and Phe147. The B ring is also shown to form two pi–pi interactions with Phe104 and Phe147, whereas Tyr102 forms pi–pi interactions with the A ring. Furthermore, pi interactions were observed between the C ring of isorhamnetin and Leu61 and Ile117, with one between the B ring and I63, L71, L74 and V113, as shown in the two-dimensional plots of Figure 5b,c. Therefore, isorhamnetin displays extensive hydrophobic interactions with the hydrophobic pockets of MD-2, resulting in a tight binding affinity. Furthermore, 7–OH of isorhamnetin forms a hydrogen bond with Tyr102. It was reported that xanthohumol and curcumin also exhibit the same interactions with Tyr102 of MD-2 [23,24]. Docking studies and mutation studies of R90A and Y102A show that Arg90 and Tyr102 are the crucial residues required for the recognition process during inhibitor binding to the MD-2 protein [25,26,27]. These results confirm that Tyr102 might be the crucial residue required for binding between isorhamnetin and MD-2.

## 3. Discussion

Isorhamnetin, a natural flavonoid and metabolite of quercetin, has gained much scientific attention due to its broad range of biological activities. Specifically, it is reported to exert anti-inflammatory [19], anti-gastric cancer [28] and antioxidant [17,29] effects. In a recent study carried out by Yang et al., it is found that isorhamnetin can protect against LPS-induced acute lung injury by inhibiting cyclooxygenase-2 (COX-2) expression [30]. However, to the best of our knowledge, there have been no studies reporting the efficacy of isorhamnetin in protecting mice from bacterial sepsis. Therefore, we explored this aspect in the present study.

Natural products isolated from plants and herbs are categorized in complementary and alternative medicine, and are now replacing synthetic drugs. A report published by the National Health Interview Survey (NHIS) reveals that approximately 38% of adults in America use complementary and alternative medicine [31], which shows the importance of natural product research. Among these natural products, flavonoids have gained increasing attention due to their wide range of biological activities [9,11]. Flavonoids are reported to exert anticancer effects by inducing apoptosis in cancer cells and interfering with signal transduction pathways associated with metastasis [32]. Quercetin, a well-studied flavonoid found abundantly in apples, berries and *Brassica* vegetables, is reported to stabilize mast cells and protect the gastrointestinal tract; moreover, its potent anti-inflammatory and antioxidant activity was also reported [33]. However, the toxicity and efficacy of these compounds have always been an obstacle for their development as alternative medicines. Therefore, we examined the toxicity of isorhamnetin using a mouse model, in which isorhamnetin was administered alone without bacteria, and evaluated the AST, ALT and BUN levels. We found that this compound does not alter these biochemical parameters, suggesting that it is safe to use as a drug. In contrast, in our previous study, rhamnetin, another derivative of quercetin, was found to exhibit anti-inflammatory activity by binding to c-Jun NH2-terminal kinase 1 and p38 MAP kinase [34]; however, it exhibits cytotoxicity towards HEK293 cells. In another study, we report that tamarixetin, another derivative of quercetin, exhibits anti-septic activity similar to that of isorhamnetin [35].

The exaggerated production of cytokines is a major cause of systemic inflammation, often referred to as septic shock. Septic shock or sepsis is the most fatal condition, and here the mortality rate is predominantly high with very few treatment options [3]. As we observe that isorhamnetin protects macrophages from LPS-induced pro-inflammatory cytokine secretion, we next sought to examine the efficacy of isorhamnetin against *E. coli*-induced sepsis using a mouse model. We observed a significantly high survival rate in mice treated with isorhamnetin compared with that in the *E. coli* only group. Moreover, the bacterial load in the visceral organs (lung, liver and kidney) is greatly decreased in the isorhamnetin-treated group, suggesting that this compound protects mice from *E. coli* infection. Further, the secretion of IL-6 and TNF-α is reduced significantly in the sera and lung lysates from the isorhamnetin-treated mice, whereas this is predominantly high in the *E. coli* only group, suggesting that isorhamnetin suppresses *E. coli*-induced inflammation in mice.

To induce systemic inflammation or sepsis, bacteria or their byproducts target TLR signaling, and more specifically, the TLR4 pathway. Natural compounds, and precisely, flavonoids, represent principal candidates that target the TLR4 pathway. For example, epigallocatechin gallate, a polyphenolic flavonoid found in green tea, has been identified as a potent antagonist of the TLR4 pathway [36]. Therefore, flavonoids have always attracted research attention as valuable resources for the development of TLR4 antagonists due to their lack of toxicity and regular consumption in food. Curcumin, for example, has been explored for its various biological effects, including anti-inflammatory activity [37]. Further, cinnamic acid and its derivatives are known for their anti-inflammatory properties [25]. Similarly, cordycepin, a traditional oriental medicine from the *Cordyceps* species, is found to suppress LPS-induced inflammatory signaling by inactivating the MAPK and NF-κB pathways and inhibiting TLR4-mediated signaling [38].

In addition, curcumin, sulforaphane, 6-shagaol, glycyrrhizin, isoliquiritigenin, caffeic acid phenethyl ester, cinnamaldehyde, paclitaxel, morphine, naloxone, chitohexose and xanthohumol are natural small molecular inhibitors that target TLR4 activation and signaling [36]. Among these, xanthohumol and curcumin were further studied for their TLR4 antagonistic activity; it is reported that they interact with MD-2 with high affinity, thus interfering with the binding between LPS with MD-2, which eventually antagonizes TLR4 [23,39].

In this study, we examined the binding efficacy of isorhamnetin to MD-2 in order to regulate the TLR4/MD-2 interaction, which eventually leads to the induction of TLR4 pathway. We carried out the SEAP assay using HEK-Blue™-hTLR4 cells, as the translocation of NF-κB from the cytosol to the nucleus tends to release the SEAP protein in cell supernatant, and is directly associated with the activation of the TLR4 pathway. From this experiment, we observed that isorhamnetin treatment reduces the activation of TLR4 pathway as the secretion of SEAP protein is drastically reduced even at the 1 μM concentration, suggesting that isorhamnetin may inhibit TLR4-inflammatory signaling pathway.

Isorhamnetin, a flavonoid, has potent anti-inflammatory potential [21]; therefore, we sought to examine the target through which it executes its anti-septic activity. To examine this mechanism, we determined the binding affinity of isorhamnetin for MD-2, as the latter is responsible for TLR4/MD-2 dimerization, thereby activating the TLR4 pathway upon exposure to LPS [40]. The SPR method that we applied to examine binding reveals that isorhamnetin has micromolar binding affinity for the MD-2 protein. We further confirmed our results through docking simulations, where we observed that the hydrophobic region of MD-2 is responsible for the interaction with isorhamnetin, resulting in an antagonization of the TLR4 pathway. Especially, the O-methyl group of isorhamnetin is found to exhibit hydrophobic interactions with Leu74 and Phe147 of MD-2, and 7–OH is suggested to form a hydrogen bond with Tyr102 of this protein. Therefore, we confirmed that isorhamnetin binds directly to MD-2 in vitro and in silico.

Additionally, we carried out the LAL assay to examine whether isorhamnetin neutralizes the LPS, because that might also be another way to block the TLR4 pathway. However, our results of this in vitro LAL assay demonstrate that there is no neutralizing effect of isorhamnetin against LPS. On the other hand, there is significant difference in the amount of LPS present in the serum of the *E. coli*-infected group and the isorhamnetin-treated group, suggesting that isorhamnetin treatment exerts the reduction of the bacterial load in blood, and thus decreases the level of LPS. Taken together both in vitro and in vivo, LAL assay results indicate that isorhamnetin does not target LPS neutralization, instead it might give a competition to the LPS while binding with MD-2 in order to inhibit the TLR4/MD-2-LPS interaction which eventually downregulates the activation of the TLR4 pathway. Moreover, it is reported that treatment of isorhamnetin significantly decreases the expression of TLR4 and MyD88 in the LPS-treated cell [21]. Similarly, curcumin is reported to non-covalently interact with MD-2, and this interaction leads to competitive binding for LPS, resulting in the inhibition of TLR4/MD-2 dimerization, which is essential to initiate the TLR4 cascade [39,41]. Therefore, it can be suggested that isorhamnetin binds to MD-2, resulting in the inhibition of the TLR4/MD-2 dimerization, which may eventually downregulate the activation of TLR4 pathway-induced inflammation and sepsis.

In conclusion, this is the first study, to our knowledge, to report that isorhamnetin, a natural flavonoid, protects against *E. coli*-induced sepsis in a mouse model. Moreover, our study, for the first time, reports that from binding affinity, molecular docking and SEAP assay experiments, isorhamnetin directly interacts with MD-2, implying that this compound could be a potent therapeutic candidate for the treatment of TLR4-mediated inflammation and sepsis.

## 4. Materials and Methods

### 4.1. Chemicals and Biological Reagents

All chemicals, unless otherwise stated, were of the highest quality (this is a general statement for all chemicals used; we can not specify the exact purity) and were used as supplied. Isorhamnetin (95.0% purity) was purchased from Sigma-Aldrich (St. Louis, MO, USA). We further purified it to 99% purity using high performance liquid chromatography in Korea Basic Science Institute (Ochang, South Korea). Compounds were dissolved in dimethyl sulfoxide (DMSO) to produce a 10-mg/mL stock solution.

### 4.2. Animals

Female BALB/c (six weeks old) mice were used for the *E. coli*-induced sepsis model. All mice used in this study were purchased from Orient (Daejeon, Korea) and were housed under specific pathogen-free conditions in a temperature- and humidity-controlled environment for one week prior to the experiments. All procedures were reviewed and approved by the Institutional Animal Care and Use Committee (IACUC) of Konkuk University, South Korea (IACUC number: KU18163-1).

### 4.3. SEAP Assay

HEK-Blue™-hTLR4 cells (toll-like receptors (TLRs)), obtained via the co-transfection of human TLR4, MD-2 and CD14 co-receptor genes and an inducible SEAP reporter gene into HEK293 cells, were purchased from Invitrogen (San Diego, CA, USA). HEK293 cells that stably expressed TLR4 contained a secreted alkaline phosphatase reporter gene (SEAP) located downstream of the *NF-κB* promoter. HEK-Blue™ hTLR4 cells were seeded in 96-well plates at 2.5 × 10^4^ per well, using HEK-Blue detection media (Invitrogen; San Diego, CA, USA) and these were treated with isorhamnetin. After 1 h, LPS at 20 ng/mL was added for stimulation. After 16 h, SEAP production was determined based on the absorbance of the supernatant, measured at 630 nm, as described previously.

### 4.4. Measurement of Binding Affinity by SPR

The binding affinity between isorhamnetin and MD-2 was determined by SPR using a Biacore T100 (GE Healthcare, Sweden). Briefly, MD-2 protein was immobilized onto a CM5 chip using a standard EDS/NHS amine coupling method with sodium acetate (pH 5.0, acidic). MD-2 protein at 30 μg/mL was injected onto the surface of the CM5 chip at a resonance value of 2300. To analyze the kinetics, we used a running buffer composed of phosphate-buffered saline (PBS) (pH 8.0, slightly alkali), 0.05% Tween 20 and 1% DMSO. The chip was regenerated with 5 mM sodium hydroxide (NaOH) after the analysis of every sample. Equilibrium dissociation constant (K_D_) values for the binding affinity were measured using a 1:1 binding assay with BIA Evaluation 2.0 software (GE Healthcare, CA, USA).

### 4.5. Molecular Docking

To analyze the TLR4/MD-2/isorhamnetin interaction, molecular docking was carried out using AutoDock Vina implemented by Yasara software (http://www.yasara.org) [42]. The crystal structure of the human TLR4/MD-2/*E. coli* LPS Ra complex was obtained from the protein data bank (PDB ID: 3FXI). The structure of isorhamnetin was acquired from the PubChem compound database (PCID:5281654), and the structure was subjected to energy optimization before docking. Water molecules and hetero atoms were removed from the protein, and polar hydrogen atoms were added. One hundred docking cycles with 500,000 evaluation steps were performed. Based on the cluster information, binding energy, molecular interactions such as hydrogen bonds and hydrophobic interactions, the best docked pose was selected.

### 4.6. Survival test for the sepsis mouse model

Survival tests were carried out as described previously [43]. Briefly, 20 BALB/c mice were used in each of the four groups. For the control group, mice were administered an intraperitoneal (i.p.) injection of PBS. For the second group, mice were administered an i.p. injection of 0.2 mL of *E. coli* (1 × 10^7^ CFU/mouse). For the third group, mice were administered an i.p. injection of isorhamnetin (1 mg/kg) with 0.2 mL of *E. coli* (1 × 10^7^ CFU/mouse). For the fourth group, mice were administered an i.p. injection of isorhamnetin (1 mg/kg) without *E. coli*. Survival in each group was observed for up to four days (0, 6, 12, 18, 24, 30, 36, 48, 60, 72, 84 and 96 h).

### 4.7. Cytokine Levels in the Serum and Lung Lysates in the Sepsis Mouse Model

Isorhamnetin (1 mg/kg) was injected 60 min before the *E. coli* (1 × 10^7^ CFU/mouse) injection, and the mice were housed for 12 h. After 12 h, the mice were sacrificed, and blood and lung tissues were collected to examine inflammatory cytokines (TNF-α and IL-6) in the serum or lung lysates, using respective ELISA kits (R&D system), according to the manufacturer’s instructions.

### 4.8. Detection of AST, ALT, and BUN in the mouse serum

The detection of aspartate aminotransferase (AST), alanine amino transferase (ALT) and blood urea nitrogen (BUN) in the serum was performed using a standard kit available from Asan Pharmaceutical, (Gyeonggi-do, South Korea) as per the manufacturer’s instructions. The levels of AST, ALT and BUN were determined relative to a standard provided by the kit after subtracting the background levels. PBS was used as the negative control and a standard solution was used as the positive control to calculate the rates.

### 4.9. Determination of E. coli Counts in Organ Tissues

At the time of sacrifice, the lungs, liver and kidneys were removed aseptically and placed separately in 1 mL of sterile PBS. The tissues were then homogenized on ice using a tissue homogenizer under a vented hood. The lung, liver and kidney homogenates were diluted with PBS to 1:1000. After plating 10 µL of each diluted sample onto Luria Bertani (LB) agar, the plates were incubated at 37 °C for 24 h. We then counted the numbers of *E. coli* colonies, which were used to assess the relative abundances of *E. coli*.

### 4.10. LAL Assay

To determine the endotoxin concentration in mice serum we have carried out the LAL assay. The assay was carried out using Pierce LAL Chromogenic Endotoxin Quantification Kit (Thermo Scientific, Rockford, IL, USA) according to the manufacturer’s instruction. Similarly, to examine the LPS neutralizing efficacy of isorhamnetin in vitro, we incubated the different concentrations of isorhamnetin (0, 1, 5, 10, 25 and 50 μM) with 1ng/mL of LPS at 37 °C in a 96-well plate which was then analyzed by a Pierce LAL Chromogenic Endotoxin Quantification Kit (Thermo Scientific, Rockford, IL, USA) according to the manufacturer’s instruction.

### 4.11. Histopathological Examination

For histopathological examination, all samples were fixed in 10% formalin buffer and dehydrated with graded alcohol. The tissues were then embedded in paraffin blocks, and pathological sections were sliced along the longitudinal axis. From each sample, 5-mm thick sections were obtained, and staining with hematoxylin and eosin was performed to evaluate lung tissue morphology.

### 4.12. Statistical Analysis

All statistical analyses were carried out using GraphPad Prism software. Dunnett’s multiple comparisons test (Prism 7.0, GraphPad Software Inc., La Jolla, CA, USA) was used for the comparisons of multiple groups. Values were considered statistically significant at *p* < 0.05. The error bars represent the mean ± standard error of the mean (SEM) (* *p* < 0.05, ** *p* < 0.01, and *** *p* < 0.001 compared to cells treated with agonist).
